# High protein diet is of benefit for patients with type 2 diabetes: An updated meta-analysis: Erratum

**DOI:** 10.1097/MD.0000000000013754

**Published:** 2018-12-14

In the article, “High protein diet is of benefit for patients with type 2 diabetes: An updated meta-analysis”,^[1]^ which appears in Volume 97, Issue 46 of *Medicine* the authors’ degrees appear incorrectly and should be:

Zhao Wen-Ting, MS; Luo Yu, MD; Zhang Ying, MS; Zhou Yun, BD; Zhao Ting-Ting, BD.
